# Integrated morphological analyses of *Cladomorphus phyllinus* and transcriptomic analysis of *Cladomorphus trimariensis* provide insights into the cardiac morphophysiology of stick insects (Phasmida: Phasmatidae)

**DOI:** 10.1007/s00441-026-04084-3

**Published:** 2026-06-23

**Authors:** Vinícius Cordeiro Rocha, Henrique Barbosa da Silva, Renata Cristina Barbosa, Gustavo Ferreira Martins

**Affiliations:** Departamento de Biologia Geral, Universidade Federal de Viçosa, Viçosa, Minas Gerais 36570-900 Brazil

**Keywords:** Circulatory system, Transcriptomics, Dorsal tube, Immunity, Pericardial cells, Hemocytes

## Abstract

The heart of Phasmida (stick insects) remains poorly understood, particularly regarding its overall physiology and associated cells. Here, we integrate the morphology of the heart and associated cells in *Cladomorphus phyllinus* with transcriptomic profiling of corresponding structures in *Cladomorphus trimariensis*. The heart is a tubular structure composed of cardiomyocytes physically associated with alary muscles, with a location and structure similar to that described in other insects. Numerous pericardial cells are organized in cord-like aggregations around the heart, displaying abundant membrane invaginations and an extensive endocytic and lysosomal machinery, consistent with their role in hemolymph filtration. Given their abundance, these cells likely represent the main source of the numerous transcripts related to endocytosis, vesicular trafficking and lysosome-mediated digestion. Hemocytes are also present around the heart, often forming aggregations indicative of immune responses. The transcriptomic analysis indicates immune activity, including melanization and antibacterial defense, predominantly driven by the hemocytes, with the pericardial cells providing a supportive role. Our results confirm that heart and associated cells act not only in hemolymph propulsion, but also as a site involved in maintaining hemolymph homeostasis and immune defense. This work provides an integrated morphological and functional framework for the circulatory system of Phasmida, expanding comparative perspectives across insects.

**Supplementary Information:**

The online version contains supplementary material available at 10.1007/s00441-026-04084-3.

## Introduction

Over 3500 species of stick insects (Phasmida) have been described to date, mostly in tropical or temperate regions (Bradler and Buckley 2018, Brock, et al. 2022). Most species display a nocturnal behavior, feeding on a wide variety of plants, with a few causing substantial crop damage due to intense feeding that results in tree defoliation (Baker 2015; Bedford 1978; Liu 2021).

*Cladomorphus* stick insects are native to Brazil, displaying a large size and resembling dry sticks (Alvarenga, et al. 2018). They feed on the leaves of several plants, such as *Psidium guajava* (guava) and *Piptania* sp. (angico). These insects display a high sexual dimorphism, with the adult females being wingless and larger (~ 23 cm) when compared to males (~ 13 cm) (Alvarenga, et al. 2018). *Cladomorphus phyllinus* Gray is the most commonly found Brazilian species, kept in museums and universities for scientific and educational purposes (Torres, et al. 2022). *Cladomorphus trimariensis* Kumagai and Fonseca is a highly similar species, first described in Brazilian savannas (Kumagai and Fonseca 2009). The biology of *Cladomorphus* has been considerably understudied, with the investigation of its digestive tract being one of the few exceptions (Azevedo, et al. 2013). Similar to several groups of insects, no attention has been given to the circulatory system of these insects.

The insect circulatory system transports nutrients, hormones, neuropeptides, immune factors and metabolic waste throughout the body cavity, having the dorsal vessel as the central component. The dorsal vessel is a muscular tube extending from the head to the abdomen, divided into an anterior aorta, located in the head and thorax, and a posterior heart, located in the abdomen (Chiang, et al. 1990; Hillyer and Pass 2020). Hemolymph enters the heart through incurrent ostia, being propelled along its lumen by rhythmic muscle contractions. In general, the hemolymph is released at the anterior end of the dorsal vessel in the head and by excurrent ostia, with a localization along the dorsal vessel varying across insects (Angioy, et al. 1999; Ejaz and Lange 2008; Hillyer 2018; Hillyer and Pass 2020).

The wall of the insect’s heart is formed by contractile cardiomyocytes (Barbosa da Silva, et al. 2019; Hillyer and Pass 2020; Leódido, et al. 2013; Souidi and Jagla 2021). Alary muscles support the organ throughout its length, maintaining the structural integrity, anchoring it to the body wall, and facilitating heart expansion (Chiang, et al. 1990; Martins, et al. 2011; Rugendorff, et al. 1994).

Pericardial cells and hemocytes are commonly found in close associations with the heart, with a number and organization varying across insects (Barbosa da Silva, et al. 2019; Crossley 1972; Curtis, et al. 1999). Pericardial cells are regulators of hemolymph composition through filtering and intracellular degradation (Crossley 1972; Fife, et al. 1987; Mills and King 1965), while hemocytes perform humoral and cellular immune responses against foreign bodies or pathogens from the hemolymph (Hillyer and Strand 2014; Sigle and Hillyer 2018). Together, the heart and associated cells form a structurally integrated unit, composed of cells that complement the heart’s function as the organ responsible for hemolymph propulsion, either by providing mechanical support or by performing activities that take advantage of the hemolymph flow in this region.

One of the main reasons for investigating the insect’s heart physiology comes from its physiological similarities with the vertebrate heart, as demonstrated in studies with *Drosophila* flies, enabling those to be models for studies of heart development, ageing and cardiac disorders (Bodmer and Venkatesh 1998; Souidi and Jagla 2021; Zhu, et al. 2017). Nevertheless, several features of heart morphophysiology in insects still remain to be elucidated, partly because this complex structure has been largely neglected in studies, but also due to the challenges of sample isolation and preparation, given that the organ is relatively thin, fragile and attached to the body wall by alary muscles.

The heart of Phasmida has been investigated in *Baculum extradentatum*, and morphological analyses revealed a tubular organ composed of sequential chambers, extending through the nine abdominal segments. Incurrent ostia are located between these chambers, while excurrent ostia are present in the first two abdominal segments. Nine pairs of alary muscles are situated at the intersegmental junctions of the abdomen, supporting the heart on both sides and anchoring it to the body wall (Ejaz and Lange 2008).

The heart of Phasmida insects still remains largely unexplored, not only in the functional scale but also when it comes to the organization and ultrastructure of associated cells. A deep investigation into the heart of these species, facilitated by their large size, broadens the understanding of the insect circulatory system and its functional organization. This study addresses a major gap in phasmid biology by integrating morphological and whole-transcriptome (RNA-seq) analyses of the heart and associated cells in two closely related species (*C. phyllinus* and *C. trimariensis*). It sheds light on the structural organization and physiological profile of the phasmid heart and its associated cells, and demonstrates how this organ integrates circulatory function with innate immune processes, offering new insights into the integration of circulation, immunity, and homeostasis in insects.

## Material and methods

### Insects and dissection

*Cladomorphus phyllinus* specimens were provided by the Departmento de Entomologia and Acarologia at Escola Superior de Agricultura Luiz de Queiroz – Universidade de São Paulo, São Paulo. *Cladomorphus trimariensis* specimens were provided by the Laboratório de Fisiologia e Neurobiologia de Invertebrados, at Universidade Federal de Viçosa, Viçosa, Minas Gerais, Brazil. Both species were kept in plastic cages without control of temperature or photoperiod, fed daily with guava leaves ad libitum. Two species were used because the colony of *C. trimariensis* was lost after the transcriptomic analysis conducted in 2014, making it unavailable for the morphological analyses performed in 2025 and 2026. Because these species are closely related, we integrated and interpreted morphological and transcriptomic data across species, as it was not possible to use the same species for both morphological and gene expression analysis.

The insect abdomen was cut laterally along the pleura using micro-scissors, after which the digestive and reproductive systems were removed. The dorsal abdominal region containing the heart and associated tissues was maintained in PBS and imaged fresh using a Canon EOS Rebel T7i camera coupled to a stereomicroscope for a first-instar nymph, whereas a Samsung S23 smartphone camera was used for adult female *C. phyllinus*. The heart and associated cells were then grasped at its posterior end (last abdominal segment) and gently pulled out with forceps. Prior to fixation, fat body cells were carefully removed with forceps, and the organs were transversely sectioned with a micro-scissor into smaller fragments to enable analyses.

### Light microscopy

The heart and associated cells from three nymphs from *C. trimariensis* (3rd instar), three immature specimens (1st instar) and four adult females of *C. phyllinus* were dissected in phosphate-buffered saline (PBS, 0.1 M, pH 7). Tissues were fixed in a Zamboni’s fixative (paraformaldehyde 4% and picric acid 0.4% in PBS) for 2 h and then rinsed in PBS. To enhance membrane and lipid preservation in semithin sections, samples were post-fixed in 1% osmium tetroxide in 0.1 cacodylate buffer (pH 7.2) (Electron Microscopy Sciences, Hatfield, PA, US) (0.1 M) for 2 h, followed by PBS rinses. Samples were dehydrated in a graded ethanol series (70–100%), 10 min in each concentration, and infiltrated with a mixture of anhydrous ethanol and Leica Historesin (2-hydroxyethyl-methacrylate, Leica Microsystems, Heidelberg Mannheim, Germany) for 1 h. They were then transferred to pure historesin for 24 h and subsequently embedded in fresh historesin containing catalyst (15:1) for polymerization.

Sections (3–5 µm) were obtained using a Reichert Jung 2050 microtome with glass knives. The sections were then stained with toluidine blue for 30 s, or Giemsa (20 min) or Mercuric Bromophenol Blue (2 h) (for protein detection). The stained sections were then washed, dried and mounted with Entellan (Sigma-Aldrich, Missouri, USA). Slides were analyzed and photographed using an Olympus BX53 or an Opticam O600R light microscope.

### Transmission electron microscopy (TEM)

The heart and associated cells from two *C. phyllinus* adult females were dissected in PBS. Small tissue fragments were fixed in 2.5% glutaraldehyde in 0.1 cacodylate buffer containing sucrose (pH 7.2) solution (sucrose/cacodylate buffer, 0.1 M, pH 7.2) for 2 h and rinsed in PBS. Samples were post-fixed in 1% osmium tetroxide (0.1 cacodylate buffer, pH 7.2) for 2 h, followed by PBS rinses.

Tissues were dehydrated in a graded ethanol series (70–100%) and infiltrated in a 1:1 mixture of absolute ethanol + LRWhite resin (London Resin Company Ltd, Berkshire, UK) for 24 h. They were then transferred to pure LRWhite for an additional 24 h period, embedded in fresh resin in gelatin capsules (0.95 mL) (Electron Microscopy Sciences, Hatfield, PA, USA), and polymerized at 60 °C for 24 h. Ultrathin sections were obtained with an RMC Power Tome-X ultramicrotome, contrasted with 3% uranyl acetate and 0.2% lead citrate, 20 min each, and examined with a Zeiss EM 109 transmission electron microscope at Núcleo de Microscopia e Microanálises (NMM-UFV).

Micrographs (light microscopy and TEM) were processed using image-editing software, including Photoshop and GIMP, to improve contrast and, when necessary, remove noise or artifacts. Image adjustments were kept to a minimum to avoid interfering with data interpretation.

### RNA-seq analysis

#### RNA extraction and sequencing

Hearts and associated cells were dissected from six adult males of *C. trimariensis*, transferred to TRIzol reagent (Thermo Fisher Scientific Inc., Waltham, Massachusetts, USA) and stored at − 80 °C until extraction. Total RNA was isolated following the manufacturer’s protocol (Invitrogen, Thermo Scientific Inc.), including DNase I treatment for removal of genomic DNA for 15 min. A total of 400 ng of RNA was diluted to a final volume of 400 µl with nuclease-free water.

RNA quality was initially assessed by agarose gel electrophoresis following Bryant and Manning (1998). RNA integrity was evaluated using an Agilent 2100 Bioanalyser with RNA 6000 Nano chips (Agilent, Waldbronn, Baden-Württemberg, Germany) at the Integrated Genomics Facility from Kansas State University (KSU, Manhattan, KS, USA). RNA quantification was determined with a Qubit 2.0 Fluorometer (Thermo Fisher Scientific), and purity (A260/280 and A260/230 ratios) was measured using a NanoDrop ND-1000 spectrophotometer (Thermo Fisher Scientific).

RNA-seq libraries were prepared with 400 ng of high-quality RNA using the TruSeq RNA Sample Preparation Kit v2 (Illumina), according to manufacturer’s instructions. Paired-end sequencing (2 × 300 bp) was performed on an Illumina MiSeq platform using a MiSeq Reagent Kit v3(600 cycles) at the KSU Integrated Genomic Facility.

Access to the genetic resources was registered in SISGEN (National System for the Management of Genetic Heritage and Associated Traditional knowledge) under license #A0928E6. Raw data and metadata are available in National Center for Biotechnology Information (NCBI) under BioProject ID PRJNA1339138 and BioSample accession SAMN52625896.

#### Bioinformatics

Raw reads were quality-checked using FastQC tool (Andrews 2010) (v0.11.5). The low-quality reads and short reads along with adapter remnants were trimmed out using Trimmomatic (Bolger, et al. 2014) (v0.36). The trimmed reads were then submitted to kraken2 (Wood and Salzberg 2014) (v2.0.8) for removal of exogen contaminants, followed by a new quality-checking step using FastQC.

The transcriptome assembly was performed using Trinity (Grabherr, et al. 2011; Haas et al. 2013) (v2.15.1), following a de novo approach (Raghavan, et al. 2022), since there is no reference genome available for this species. The final assembly was quality-checked using Salmon (Patro, et al. 2017) (v1.10.1), measuring the alignment of the trimmed reads to the final assembled transcriptome. Moreover, the final assembly was also submitted to BUSCO (Benchmarking Universal Single-Copy Orthologs, v5.7.0, busco.ezlab.org) for the analysis of expected orthologs according to the database insecta_odb10. The assembled transcriptome was submitted to CD-HIT (Fu, et al. 2012) (v4.8.1), in order to decrease redundancy in the transcripts. Following this, the open-reading frames (ORFs) were identified in the assembly using TransDecoder (Haas et al. 2013) (v5.7.1).

The identified ORFs were submitted to Salmon for analysis of expression through transcript per million (TPM) counts. For ORF annotation, two separate tools were employed, EggNOG-mapper (evolutionary genealogy of genes: non-supervised orthologous group) (Huerta-Cepas, et al. 2017) (v2.1.8) and Diamond (Swisspro/Uniprot) (Buchfink, et al. 2021) (v2.1.13). The cluster of orthologous groups (COG) and gene ontology (GO) terms associated with each one of the annotated ORFs were obtained by EggNOG-mapper. The amino acid sequences corresponding to the ORFs that displayed no hits against any of the annotation tools utilized were further investigated through InterProScan (Quevillon, et al. 2005) (v5.59–91.0).

## Results

### General anatomy and histology of the heart of C. phyllinus

**Fig. 1:**
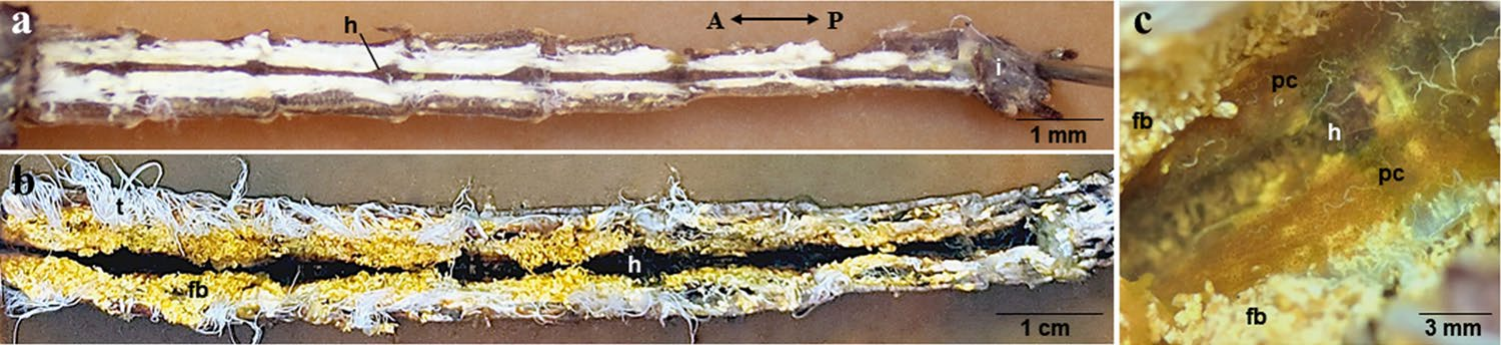
Internal view of the dorsal abdominal region of a first-instar nymph (**a**) and an adult female (**b**, **c**) of *Cladomorphus phyllinus*. The heart (h) is translucent and extends through all abdominal segments. A–P: anteroposterior body axis;** c** Abdominal segment showing the transparent heart (h) with a mass of beige-yellow pericardial cells (pc); fb: fat body; i: integument; t: trachea

**Fig. 2:**
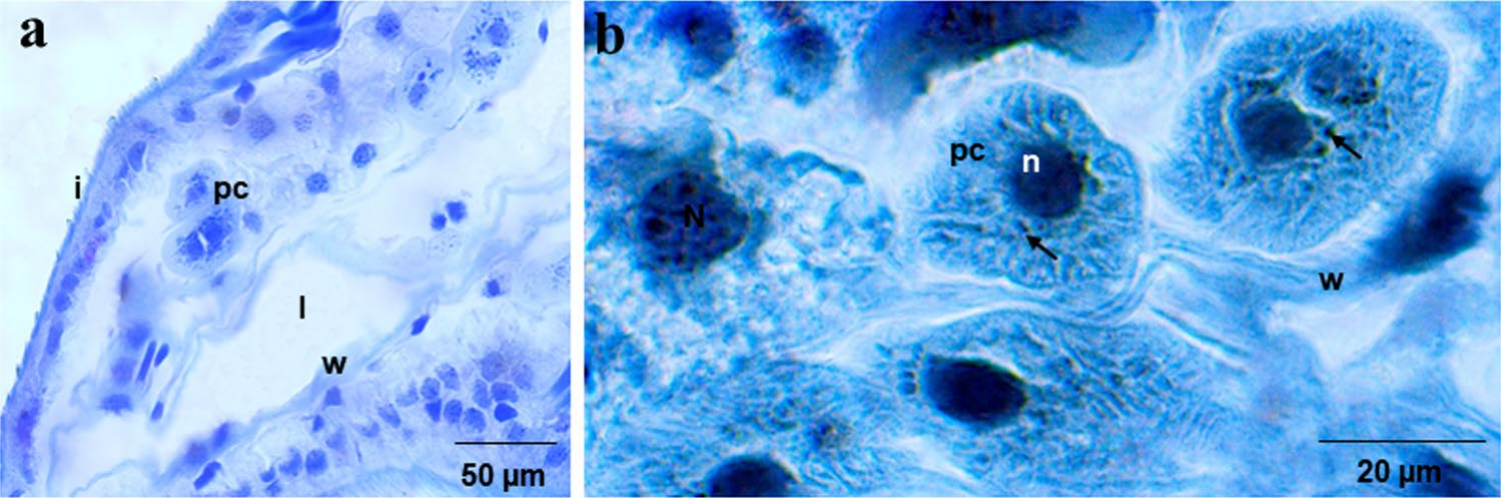
Histological sections of the heart of a *Cladomorphus phyllinus* first-instar nymph. **a** Heart (w) and pericardial cells (pc) stained with Giemsa. **b** Pericardial cells showing intensely stained perinuclear inclusions (arrows) stained with mercuric bromophenol blue. i: integument; l: heart lumen; n: nucleus; w: heart wall

**Fig. 3:**
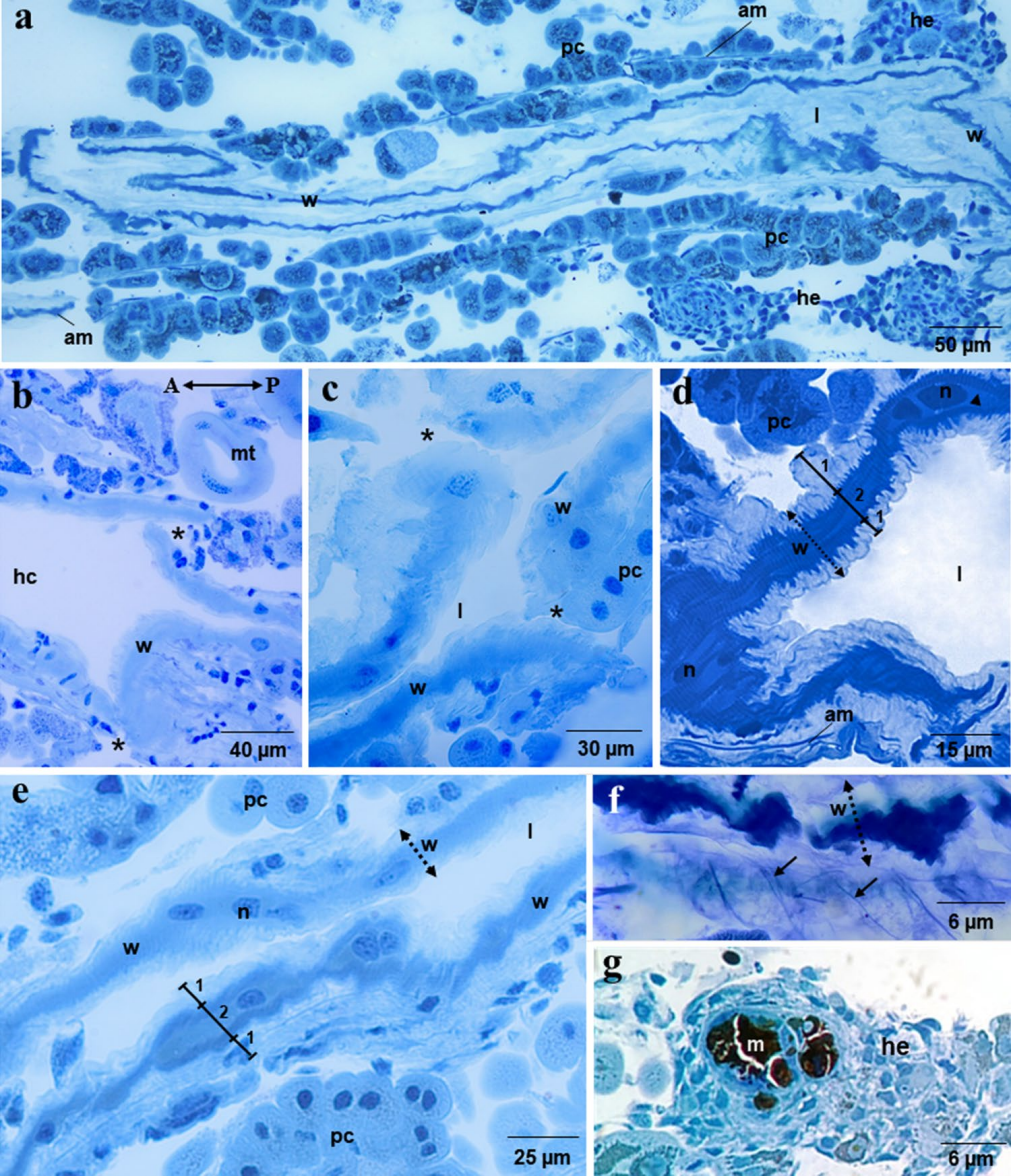
Histological sections of the heart of an adult female *Cladomorphus phyllinus* stained with toluidine blue (**a**, **d**, **f**, **g**), Giemsa (**b**), and mercuric bromophenol blue (**c**, **e**). **a** Longitudinal section of a portion of the heart showing numerous pericardial cells (pc) in close proximity, organized in cord-like aggregations associated with alary muscle ramifications (am). Hemocyte aggregations (he) are observed near the pericardial cells. **b** and** c** Longitudinal sections showing paired openings (*) in the heart wall (w), with **b** showing putative incurrent ostia and **c** putative excurrent ostia. **d** Heart wall (w) with multinucleated cardiomyocytes displaying a dual staining pattern: weakly stained peripheral regions (1) (the inner region facing the lumen and the outer region in contact with the pericardial cells), and a more intensely basophilic region (2) in the inner portion of the muscle fiber, highlighting the striated myofibrils. Near the cardiomyocyte nuclei (n), striations are not evident, and staining appears homogeneous (arrowhead). **e** Longitudinal section of the heart wall with cardiomyocytes stained with bromophenol blue, displaying the dual staining pattern observed in **d**, with region (2) positive for proteins. **f** Alary muscle ramifications (arrows) attached to the heart wall (w). **g** Hemocyte aggregation (he) associated with melanized material (m). l: heart lumen; hc: heart chamber; mt: Malpighian tubule

In nymphs and adult females of *C. phyllinus*, the heart is a transparent tubular organ extending along the dorsal region of the abdomen. The organ runs through the abdominal segments (Fig. 1a and b). In adults, masses of pericardial cells are laterally associated with the entire length of the heart (Fig. 1c). These cell masses are not readily visible in nymphs under the stereomicroscope. However, light microscopy revealed that the pericardial cells of nymphs from *C. phyllinus* (Fig. 2) and *C. trimariensis* (Fig. S1) are distributed along the lateral regions of the heart and appear, upon visual inspection, to be less numerous than those observed in adult females of *C. phyllinus*. In adult females, the heart tube is surrounded along its entire length by numerous pericardial cells forming cord-like aggregates, frequently associated with parallel ramification of alary muscles (Fig. 3a).

Ostia were identified as paired openings in the heart wall of an adult. When one extremity of the free margins of the cardiac wall was oriented toward the lumen, forming a projection resembling ostial lips, these openings were morphologically classified as incurrent ostia and were positioned between two successive cardiac chambers (Fig. 3b). Openings with the free margins of the cardiac wall oriented outward, resembling excurrent ostia, were also observed (Fig. 3c) (Pass et al. 2006; Ejaz and Lange 2008).

The heart wall is formed by cardiomyocytes arranged in a single-cell-thick layer. The cardiomyocytes are multinucleated, with nuclei located within the fiber, and exhibit a highly basophilic inner region containing the nuclei. This region stained intensely with bromophenol blue, indicating elevated protein content in adults (Fig. 3d and e). Ramifications of the alary muscles extend along the heart structure and attach to the heart wall (Fig. 3f). Distinct cellular boundaries between cardiomyocytes were not discernible.

Hemocytes were observed along the external surface of the heart. These cells formed aggregations resembling nodulation or melanization processes (Fig. 3a and g).

**Fig. 4:**
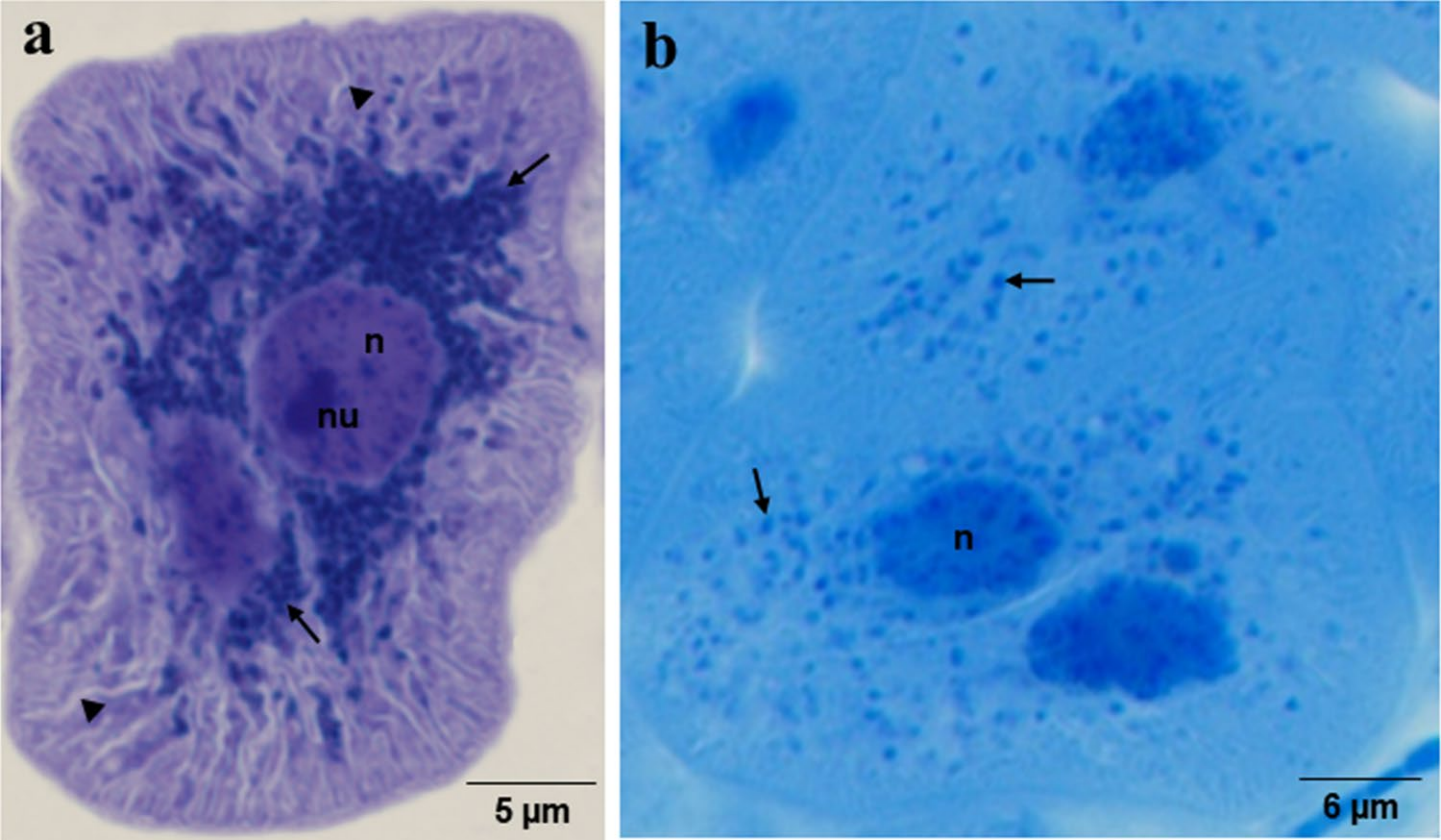
Pericardial cells of *Cladomorphus phyllinus* adult female. **a** Binucleated cell stained with Giemsa, showing invaginations (arrowheads) at the cell periphery and abundant basophilic inclusions (arrows) in the perinuclear region. **b** Cells stained with bromophenol blue showing perinuclear granules (arrows) positive for proteins. n: nucleus; nu: nucleolus

Pericardial cells are generally large, with a diameter of around 25 µm in adult females, displaying an evident nucleus (Fig. 4), with a subset of these cells appearing binucleated (Fig. 4a). In the perinuclear region, these cells contain numerous highly basophilic inclusions (Fig. 4a). A subset of these inclusions stained intensely with bromophenol blue, indicating high protein content (Figs. 2b and 4b), in both nymphs and adults. At their peripheral region, such cells display abundant membrane invaginations (Fig. 4a).

### Ultrastructure of the heart in adult female *C. phyllinus*

**Fig. 5:**
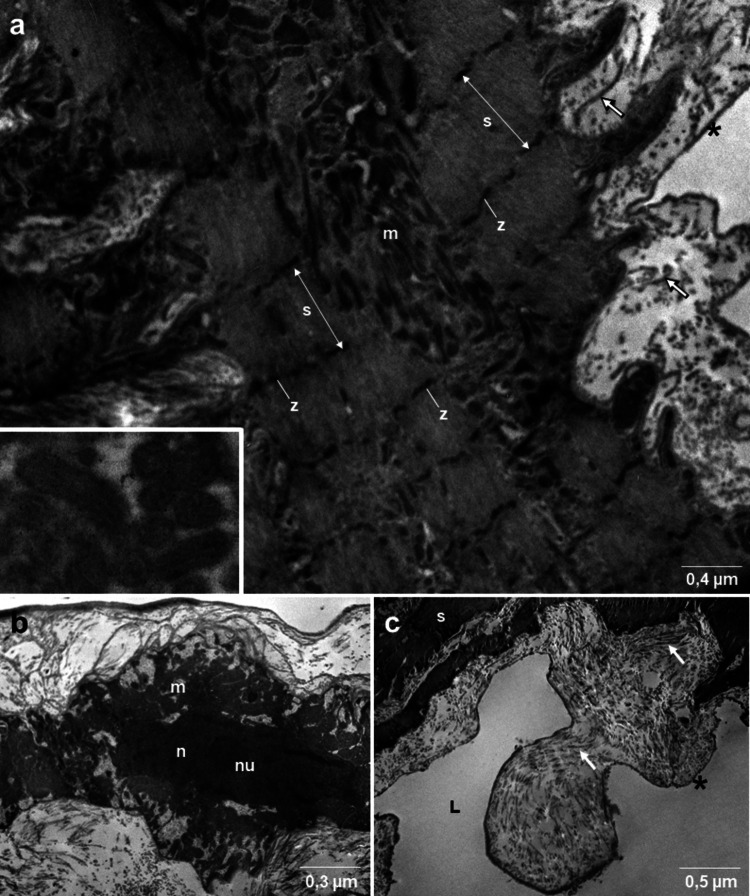
Transmission electron microscopy of the heart of an adult female of *Cladomorphus phyllinus*. **a** Longitudinal section of a portion of a cardiomyocyte containing abundant myofibrils and sarcomeres (s). z: z-line. Densely packed mitochondria (m) are visible and are highlighted in the inset. Arrows: cytoskeleton elements in the electron-lucent cell periphery. *: cell limit. **b** Cardiomyocyte containing numerous mitochondria (m) near a nucleus (n). nu: nucleolus. The periphery of the cardiomyocyte appears electron-lucent. **c** Detail of the electron-lucent cardiomyocyte periphery projecting into the heart lumen (l), with fibrous structures resembling cytoskeletal elements (arrows). *: cell limit; s: sarcomere

The heart wall of a female adult is highly electron-dense at its inner portion. This region concentrates the nuclei of cardiomyocytes and also their sarcoplasm, containing numerous mitochondria and myofibrils with evident *z*-lines. Mitochondria are concentrated in the perinuclear region (Fig. 5a and b and inset). At the electron-lucent peripheral portion, many filamentous structures resembling cytoskeletal elements were observed, although organelles were not detected (Fig. 5b and c). Alary muscles ramifications are present throughout the structure, displaying myofibrils organized within their sarcoplasm.

**Fig. 6:**
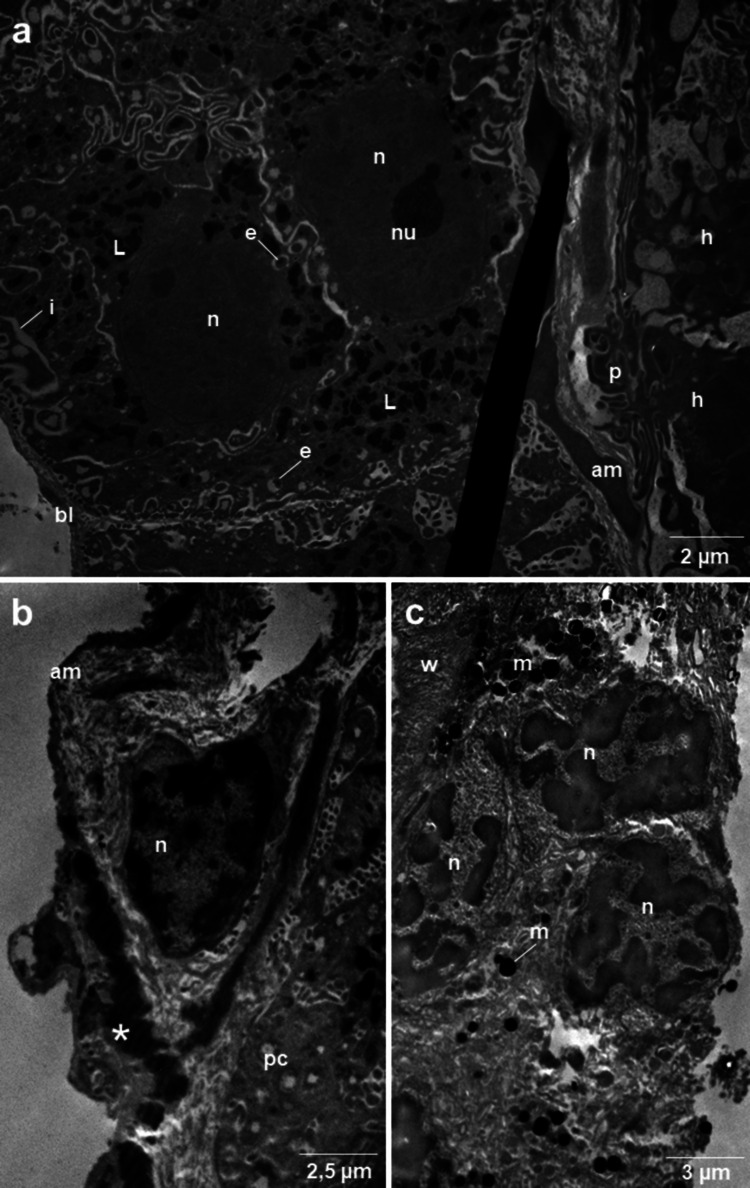
Transmission electron microscopy of heart-associated-cells of an adult female of *Cladomorphus phyllinus*. **a** Binucleated pericardial cell (pc). Numerous electron-dense lysosomes (L) are present in the perinuclear region. At the cell periphery, tubular membrane invaginations (i) or canaliculi associated with endosome-like organelles (e) are observed. am: alary muscle ramification; bl: basal lamina; h: hemocytes with pseudopodia (p); n: nucleus. **b** Portion of an alary muscle attached to a pericardial cell. *: myofibrils. **c:** Hemocyte (h) close to the heart wall (w) and containing melanized material (m) in dark granules

**Fig. 7:**
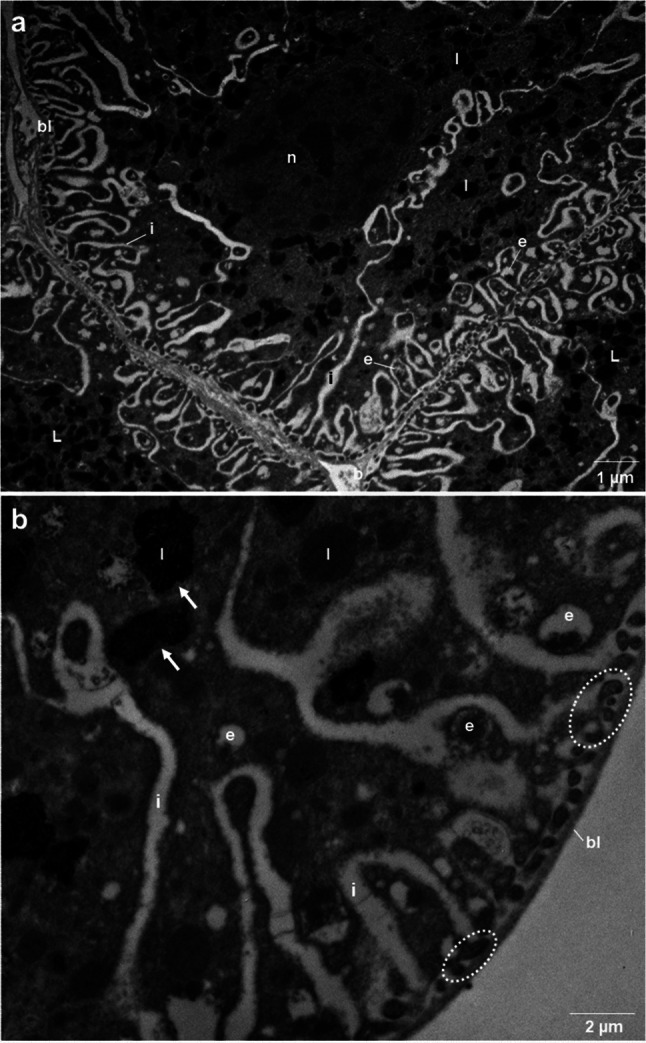
Transmission electron microscopy of pericardial cells (pc) of *Cladomorphus phyllinus*. **a** Pericardial cells in close association, enclosed by a basal lamina (bl). n: nucleus. Plasma membrane invaginations (i) extend toward the cell interior, forming canaliculi. The perinuclear region is densely packed with electron-dense lysosomes (l) and endosome-like organelles (e) often located near the canaliculi. **b** Detailed view of a peripheral region of a pericardial cell, highlighting membrane invaginations (i) associated with endosomes (e). bl: basal lamina; dotted circles: plasma membrane projections; arrow: membrane surrounding lysosomes (l)

The pericardial cells display prominent nuclei with decondensed chromatin and an evident nucleolus (Figs. 4b, 6a, and 7a). At their perinuclear region, numerous electron-dense lysosomes are present (Figs. 6a and 7a). These organelles correspond to the basophilic structures observed in Fig. 4b. At the cell periphery, tubular membrane invaginations or canaliculi are frequently associated with electron-lucent endosome-like vesicles (Figs. 6A and 7). The pericardial cells are surrounded externally by a thick basal membrane (Figs. 6a and 7). Alary muscles are often found in association to pericardial cells (Fig. 6b).

Hemocytes in proximity to the heart wall, pericardial cells or alary muscles, either isolated or in aggregates, exhibit pseudopodia and electron-dense granules resembling melanized material (Fig. 6a–c).

### Transcriptome of the heart of adult male *C. trimariensis*

#### Read processing, assembly, and completion analysis

A total of 11,585,079 reads were generated (Table 1). After the removal of low-quality reads, short reads and contaminants, a total of 10,100,707 reads remained, being utilized for transcriptome assembly. From those reads, a total of 196,135 contigs were generated by the Trinity assembler. The Salmon analysis reported an estimated total of 9,352,357 reads (92.6%) aligning to the final transcriptome assembly.

**Table 1:** Summary information for the transcriptome sequencing and analysis of *Cladomorphus trimariensis *heart and associated cells

Raw reads	11,585,079
Sequence length (bp)	70–300
Reads used for contig assembly	10,100,707
Assembled contigs	196,135
Average contig length (bp)	757
N50	1299
GC content (%)	40.23
Detected open reading frames (ORFs)	24,270

The analysis of expected orthologs in the assembled transcriptome using BUSCO (insecta_odb10) reported 1152 (84.3%) completed, 109 (8.0%) fragmented and 106 (7.8%) missing orthologs (Supplementary information. 1), fitting into the ideal range (> 80% completeness) for assemblies of non-model organisms (Raghavan, et al. 2022). The assembly thinning performed by CD-HIT-EST produced 153,514 (~ 22% reduction) representative sequences, deployed for the detection of 24,270 ORFs with TransDecoder (Supplementary information 2).

#### Transcript annotation and functional classification

**Fig. 8:**
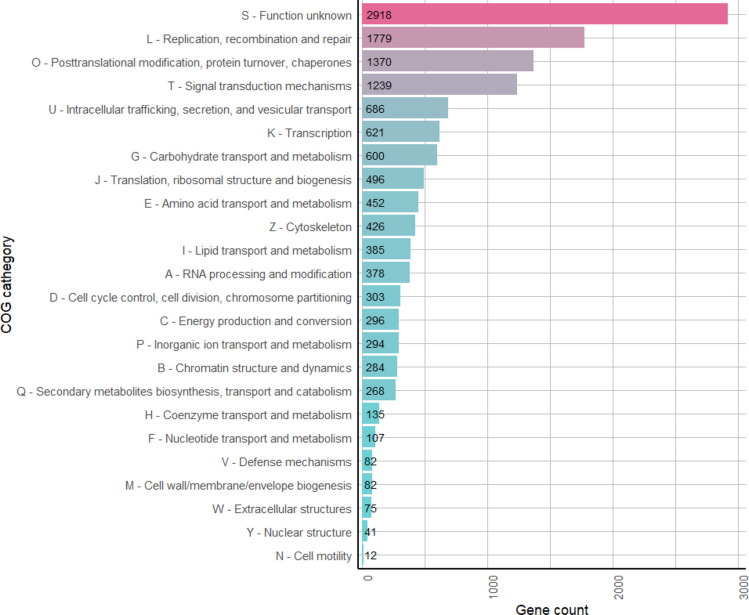
Classification of the annotated sequences from *Cladomorphus trimariensis* heart and associated cells in clusters of orthologous groups (COG) by EggNOG/Diamond, showing the number of sequences associated with each category

**Fig. 9:**
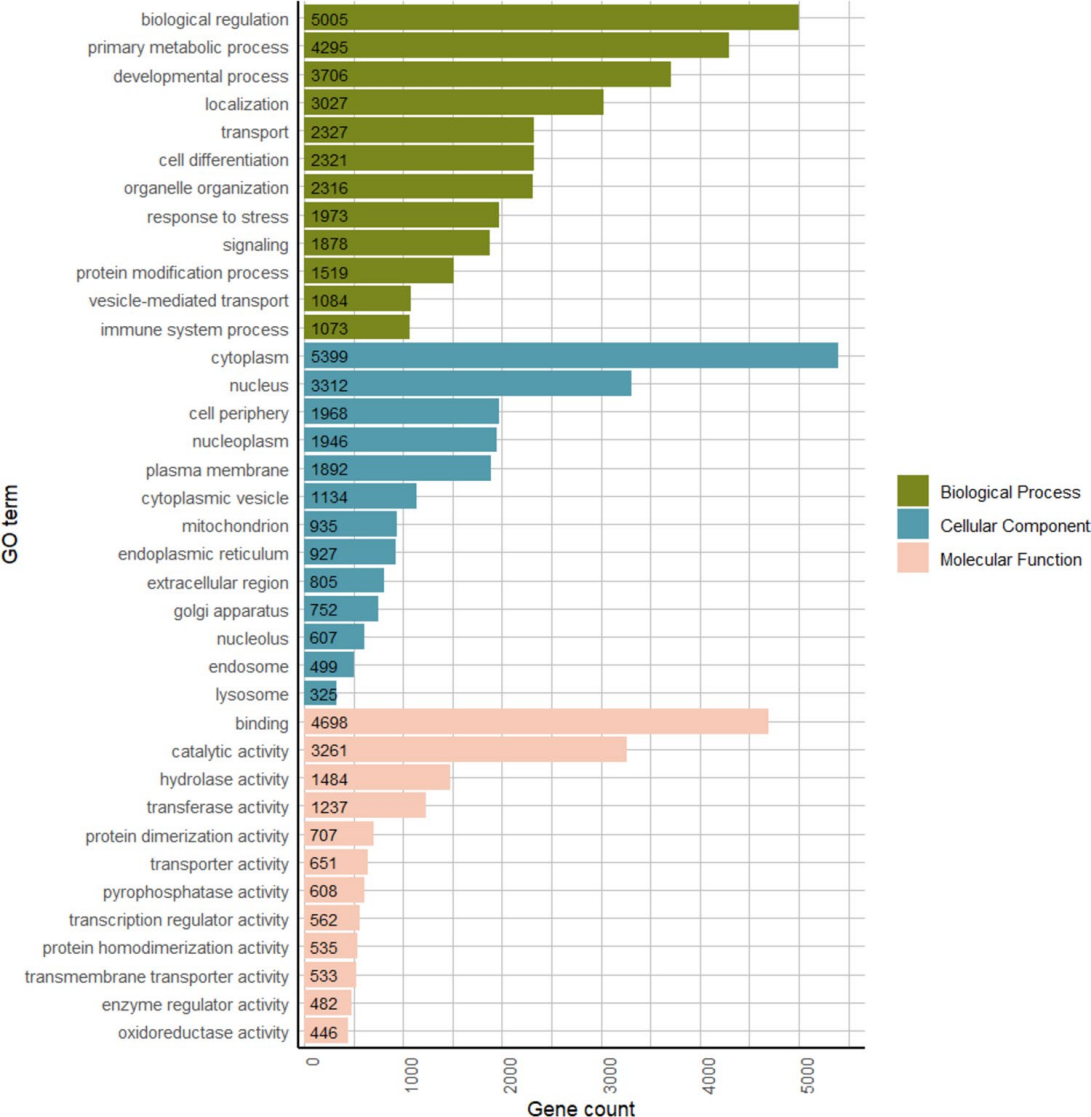
Gene ontology (GO) analysis of the sequences annotated by EggNOG/Diamond from *Cladomorphus trimariensis* heart and associated cells transcriptome. Most relevant functional categories and the number of associated sequences is shown

The ORFs were then annotated using EggNOG-mapper and Diamond (Supplementary information 3 and 4). A total of 13,833 ORFs (~ 57%) had corresponding hits (homologs) reported by the EggNOG annotation tool (minimum query and subject coverage: 50%, e-value: 0.001). The sequences annotated by EggNOG-mapper were classified in different COG (cluster of orthologous genes) categories (Tatusov, et al. 1997). The most significant categories reported were: replication, recombination and repair (1779), post translational modification, protein turnover and chaperones (1370); signal transduction mechanisms (1239); and intracellular trafficking, secretion and vesicular transport (686) (Fig. 8). A considerable number of sequences (2918) were labelled as category S (unknown) by this annotation tool. The EggNOG annotation tool also provided associated GO terms for the sequences, being classified in three main categories: cellular component, molecular function and biological process. The most relevant GO terms for each category are shown in Fig. 9.

The Salmon expression analysis (Supplementary information 5) showed that the ORFs with the highest TPM counts were those related to regular heart structure and physiology, including muscular actin (TRINITY_DN108365_c7_g1, TPM = 45,557), myosin regulatory light chain MLC-2 (TRINITY_DN4998_c1_g1, 15,552), muscle LIM protein Mlp84b (TRINITY_DN199_c0_g1, 10,272), tropomyosin (TRINITY_DN17_c0_g2, 7102) and muscle protein MP20 (TRINITY_DN108573_c1_g1, 6877). Ferritin light chain also showed very high TPM values (TRINITY_DN24407_c2_g1, 43,891) comparable to the highly expressed structural genes.

In order to investigate the role of the pericardial cells, the focus was placed on transcripts associated with the uptake and degradation of hemolymph proteins, specifically examining sequences involved in intracellular traffic and vesicular transport (COG: U, 686 sequences), as well as endosome-located (GO:0005768, 499) and lysosome-located (GO:0005764, 325) components. Based on the putative hemocyte activity around the heart, we additionally investigated transcripts associated with immunity (GO:0002376, 1073). Relevant sequences from these groups were further analyzed for expression levels using TPM estimates from Salmon (Tables 2 and 3).

**Table 2:** Annotation and expression estimates of relevant sequences related to endocytosis, vesicular transport and protein degradation within lysosomes detected in *Cladomorphus trimariensis* heart and associated cells

Trinity ID	Description (blastp)	e-value	TPM (Salmon)	Function
TRINITY_DN175_c4_g1	Ras-related protein Rab-7	4.00E-151	357	Late endosome marker
TRINITY_DN285_c0_g1	V-Type proton ATPase catalytic subunit A	0.0	155	Proton ATPase
TRINITY_DN369_c0_g1	Lysosomal aspartic protease	0.0	562	Protease activity
TRINITY_DN443_c0_g1	Vesicle-associated membrane protein 7 (VAMP 7)	8.00E-148	156	Late endosome – lysosome fusion
TRINITY_DN596_c0_g1	Cathepsin L	0.0	3685	Protease activity
TRINITY_DN1026_c0_g1	Ras-related protein Rab-5C	3.00E-155	261	Early endosome marker
TRINITY_DN1867_c0_g1	Lysosome associated membrane glycoprotein 1 (LAMP 1)	2.00E-58	445	Lysosomal membrane component
TRINITY_DN2016_c0_g1	TMEM9 family protein	3.00E-104	824	Late endosome/lysosome marker
TRINITY_DN3080_c0_g1	V-type proton ATPase subunit B	0.0	372	Proton ATPase
TRINITY_DN5951_c1_g2	Vacuolar protein sorting associated protein 37B	3.00E-113	160	Vesicular trafficking
TRINITY_DN68870_c0_g1	Clathrin heavy chain isoform X1	0.0	148	Vesicular trafficking
TRINITY_DN129790_c0_g1	Cathepsin B	0.0	768	Protease activity

**Table 3:** Annotation and expression estimates of relevant immune-related sequences detected in *Cladomorphus trimariensis* heart and associated cells

Trinity ID	Annotation (blastp)	e-value	TPM (Salmon)	Function
TRINITY_DN282_c0_g1	Lyzozyme-like	6.00E − 73	688	Defense against bacteria
TRINITY_DN638_c0_g1	Phenoloxidase-1	0.0	296	Melanization response
TRINITY_DN957_c0_g1	Akirin	2.00E − 118	126	Imd signaling pathway
TRINITY_DN959_c0_g1	Protein Toll	0.0	52	Toll signaling pathway
TRINITY_DN1434_c0_g1	Beta 1,3 glucan-binding protein	1.00E − 56	192	Pathogen recognition
TRINITY_DN1739_c0_g1	MyD88	2.00E − 116	53	Toll signalling pathway
TRINITY_DN2256_c0_g1	Nuclear factor NF-kappa-B p100 subunit	0.0	37	Imd signaling pathway
TRINITY_DN2279_c0_g1	Toll-like receptor 6	0.0	41	Toll signaling pathway
TRINITY_DN5439_c0_g1	Serine protease inhibitor 27A	0.0	66	Melanization response
TRINITY_DN10601_c0_g1	Protein pellino	0.0	14	Defense against viruses
TRINITY_DN50035_c0_g1	Holotricin-2	4.00E − 39	22	Defense against gram negative bacteria
TRINITY_DN73344_c0_g1	Phenoloxidase-activating factor-2	0.0	71	Melanization response
TRINITY_DN136987_c0_g1	Defensin	2.00E − 22	15	Defense against gram positive bacteria

#### Unknown sequences

A total of 10,437 ORFs (~ 43%) were not annotated by EggNOG/Diamond, being further investigated using InterProScan, which addresses the protein domains (Quevillon, et al. 2005). It was found significant InterPro annotations for 2878 previously unannotated ORFs (Supplementary information 6), with a considerable portion of those (22.5%) displaying zinc finger or zinc finger-like domains. The most relevant InterPro terms for the unknown proteins are shown in Table 4. A total of 7559 ORFs (31.15%) remained non-annotated.

**Table 4:** Most relevant InterPro terms for unannotated sequences

InterPro domain annotation	Sequence count
Zinc finger and zinc finger-like domains	648
Acid proteases	207
Retropepsin-like	112
L domain-like	111
Ribonuclease inhibitor	89
Leucine rich repeat	62
Ribonuclease H-like	60
Ankyrin repeat	56
LRR_typ_2	55
DNA/RNA polymerases	49
Eukaryotic and viral aspartyl proteases active site	49
ULP protease domain containing protein	49
Gag-polyprotein putative aspartyl protease	45
Integrase zinc binding domain	42
Glucocorticoid receptor-like (DNA-binding domain)	41
Immunoglobulins	38
P-loop containing nucleoside triphosphate hydrolases	32
Slit related leucine rich repeat neuronal protein	25
DNA directed RNA polymerases I, II and III subunit RPABC2	24
Fibronectin type III	24

## Discussion

The overall organization of *C. phyllinus* heart and associated cells closely matches the conserved pattern reported for most insect groups. It consists of a tubular dorsal heart composed of cardiomyocytes, supported by alary muscles, associated with numerous pericardial cells and hemocytes. This organization reflects the conservation of the circulatory system in insects, placing the heart under strong evolutionary constraint (Barbosa da Silva, et al. 2019; Helmstadter, et al. 2017; Poiani and Cruz-Landim 2007; Rotstein and Paululat 2016). Despite the large body size of *Cladomorphus*, their overall heart architecture does not display major qualitative modifications in response to increased hemolymph volumes, suggesting that such adaptations might occur at a quantitative level.

The most noticeable feature of cardiomyocytes in adults *C. phyllinus* was the contrasting pattern between an electron-lucent periphery and an electron-dense inner portion, possibly associated with the higher concentration of myofibrils and mitochondria in the inner portion, as reported for other insects (Leódido, et al. 2013; Poiani and Cruz-Landim 2006). This inner region of the cardiac fibers is more basophilic than the peripheral region and also showed a positive reaction to bromophenol blue staining, indicating a high protein content and corroborating the ultrastructural findings obtained through electron microscopy.

In the stick insect *B. extradentatum* (Ejaz and Lange 2008), both incurrent and excurrent ostia have been reported. In this species, excurrent ostia are located in the first two abdominal segments, i.e., those adjacent to the thorax, whereas incurrent ostia occur at the junction between successive heart chambers throughout the abdomen. Incurrent ostia are present at the junction of each of the chambers of the heart of *B. extradentatum*. In our specimens, however, neither the precise distribution nor the exact number of incurrent and excurrent ostia could be conclusively determined. Based on histological sections, two ostial morphotypes were identified in adults of *C. phyllinus*: one in which the margin of the muscular wall is oriented toward the lumen, classified as incurrent, and another in which the margins project outward from the organ, classified as excurrent ostia, resembling those described for *B. extradentatum* (Ejaz and Lange 2008). However, from a functional standpoint regarding hemolymph inflow and outflow, the classification of ostia in *C. phyllinus* based solely on histological sections should be interpreted with caution. Therefore, the overall organization, physiology, and functional significance of ostia in *Cladomorphus*, as well as their comparison with other Phasmida, remain to be elucidated.

At the transcriptional level, *C. trimariensis* displayed elevated TPM counts for actin, myosin and other sarcomeric components, which is consistent for a tissue rich in cardiomyocytes. Of particular interest is the Lim protein Mlp84B. RNAi-mediated silencing of Mlp84B in the heart of *Drosophila* fruit flies leads to impaired rhythmic contraction and reduced lifespan (Mery et al. 2008). These proteins likely preserve muscle structure through ensuring actin filament anchorage (Clark and Kadrmas 2013). Thus, its high number of transcripts detected here reinforces their pivotal role in maintaining the structural integrity of the heart. The protein MP20, also notably detected, was initially described in *Drosophila* synchronous muscles, being possibly a calcium-binding protein, yet its function is still poorly resolved (Ayme-Southgate, et al. 1989). Considering its elevated expression and calcium-related activity, such proteins may contribute to contraction modulation in the phasmid heart.

The GO profile of *C. trimariensis* heart shows broad similarities to the categories reported for different body parts (antennae, midgut, head, legs) from the stick insect *Clitarchus hookeri* (Wu, et al. 2016). In both species, a substantial portion of the transcripts fall within major categories such as biological regulation [GO:0065007], metabolic process [GO:0008152], signaling [GO:0023052], binding [GO:0005488], and catalytic activity [GO:0003824]. In contrast, transcripts associated with immune system processes [GO:0002376] are markedly more abundant in the heart and associated cells (this work) than in other stick insect tissues (Wu, et al. 2016).

A significant portion of transcripts could not be annotated by EggNOG or Diamond. The InterProScan analysis was used to investigate the potential functions for these ORFs based on the predicted protein domains (Mulder and Apweiler 2007). Most recovered domains corresponded to zinc-finger variants, commonly associated with DNA-binding and transcriptional regulation (Berg 1990; Brayer, et al. 2008), although some families also bind RNA or protein targets (Brayer, et al. 2008). The presence of zinc-finger domains may indicate numerous regulatory factors essential for heart functioning in Phasmida that can be potential targets for future functional studies.

A significant fraction of the transcripts remained non-annotated even after InterProScan analysis, which might be linked to the limitations of annotating for non-model organism sequences, assembled through de novo approaches. Such unknown sequences might correspond to non-functional ORFs or sequences lacking homologs in public databases, potentially representing taxonomic-restricted genes (Khalturin, et al. 2009). Furthermore, the use of restrictive parameters in the annotation pipelines, while ensuring high-quality hits, may limit the annotation of orthologs with low-sequence similarity. An elevated number of unannotated sequences has also been reported in *C. hookeri* stick insects, possibly indicating a substantial portion of the transcriptome yet to be characterized in Phasmida (Wu, et al. 2016).

Pericardial cell counts and organization vary among insects (Crossley 1972; Barbosa da Silva, et al. 2019; Leódido, et al. 2013). In *C. phyllinus*, those cells are highly abundant and organized in cord-like structures, similar to those reported for some mosquito and bees (Barbosa da Silva, et al. 2019; Poiani and Cruz-Landim 2007), an organization pattern that seems to be related to the pericardial cells anchoring themselves along the alary muscle ramifications. A high number of pericardial cells in the adults of *C. phyllinus* may constitute an adaptation to the high filtering demands imposed by a large hemolymph volume. This, alongside the apparently lower number of pericardial cells observed in nymphal stages evidences the need to investigate how pericardial cell count relates to hemolymph volume and developmental stage in Phasmida.

The occurrence of binucleated pericardial cells among insects remains a poorly understood topic. In fruit flies such cells display a single nucleus, while appearing binucleated in different mosquito species (Barbosa da Silva, et al. 2019; Leódido, et al. 2013; Rotstein and Paululat 2016). In *C. phyllinus*, two nuclei are often detected in pericardial cells; however, when cellular boundaries are not clearly discernible, it is not always possible to infer whether one or two nuclei belong to a single cell, and this number may vary among cells. How the presence of single-nucleated or binucleated pericardial cells relates to their function in hemolymph regulation still requires further investigation.

The numerous membrane invaginations in the *C. phyllinus* pericardial cells, forming canaliculi, likely increase the contact surface with the hemolymph and facilitate molecular exchange. The abundant vesicles observed near these canaliculi indicate the occurrence of endocytic processes (Brehélin and Hoffmann 1980; Brockhouse, et al. 1999; Davey 1962; Mills and King 1965). The detection of transcripts associated with endosomes [GO:0005768] and vesicular trafficking [COG: U] indicates that pericardial cells are represented in the transcriptomic profile of the *C. trimariensis* heart, reinforcing the functional interpretation of the cellular structures observed in these cells in *C. phyllinus* by TEM. The detection of clathrin and Rab family transcripts also highlights the core machinery underlying vesicle formation and trafficking (Kaksonen and Roux 2018; Martinez and Goud 1998), while the expression of early (Rab5) and late (Rab7) endosomal markers indicates the distinct stages of endosome maturation. Finally, transcripts encoding late endosome-lysosome transport proteins were also documented, including VAMP family members, suggesting trafficking towards degradative compartments (Advani, et al. 1999).

The electron-dense structures observed in the perinuclear region of pericardial cells of *C. phyllinus* suggest a lysosomal nature. This interpretation is supported by their basophilic profile, high protein content, as indicated by Giemsa and bromophenol blue staining, respectively, and pronounced electron density, characteristic features of lysosomes described elsewhere (Horobin 2011; Mazia et al. 1953). Similar patterns have also been reported in other insect species (Barbosa da Silva et al. 2019; Brockhouse et al. 1999; Poiani and Cruz-Landim, 2007). However, the use of more specific methods, such as immunostaining for lysosomal proteins, is necessary to fully clarify the nature of these structures (Yin and Yang 2024).

The perinuclear lysosomes likely degrade the content of the endocytic vesicles in the pericardial cells of *C. phyllinus* (Barbosa da Silva, et al. 2019; Brockhouse, et al. 1999). As for *C. trimariensis*, a lysosomal activity in the pericardial cells is suggested by the lysosome-located [GO: 0005764] transcripts reported in the dataset. Among those, lysosome-associated membrane proteins (LAMP), the most abundant group reported in lysosomal membranes, participating on lysosome biogenesis and phagosome-lysosome fusion (Eskelinen 2006; Huynh, et al. 2007). The transcriptome also revealed several subunits of v-type ATPases, proton pumps responsible for the maintenance of lysosomal pH gradients (Song, et al. 2020). Finally, multiple lysosomal proteases were annotated, including members of the cathepsin family, which showed consistent expression, being key mediators of intra-lysosomal protein degradation, particularly cathepsins B and L (Yadati, et al. 2020). These transcript groups were linked to pericardial cell activity based solely on the morphological analysis (this work) and available information regarding pericardial cell ultrastructure and activity from other insect species (Brockhouse et al. 1999; Hernández-Martínez et al. 2013). Therefore, their physiological function in *Cladomorphus* can only be confirmed through additional experimentation, including a differential expression analysis of pericardial cell genes.

Hemocyte aggregation around the heart was observed in several insect species, particularly at sites of intense hemolymph flow, such as the periosteal region (King and Hillyer 2012; Sigle and Hillyer 2016, 2018; Yan and Hillyer 2020). This pattern illustrates the interplay between circulation and immunity in insects, with the heart-associated hemocytes as key components of immune defense (King and Hillyer 2012; Hillyer and Pass 2020; Yan and Hillyer 2020). The hemocyte aggregations observed in *C. phyllinus* resemble the overall profile of nodulation and melanization responses, in which hemocytes surround invading particles or pathogens, ultimately leading to encapsulation (Jiravanichpaisal, et al. 2006). Moreover, the electron-dense granules present in their cytoplasm may correspond to phagocytized and melanized material. (Lavine and Strand 2002). Taken together, these observations suggest the occurrence of immune processes mediated by hemocytes in the vicinity of the heart in *C. phyllinus*, as reported elsewhere (King and Hillyer 2012; Sigle and Hillyer 2016, 2018; Yan and Hillyer 2020)*.* Nevertheless, the immune role of these cells in *Cladomorphus* lacks confirmation, which can be accomplished by performing experiments addressing how an immune challenge alters hemocyte distribution and activity around the heart.

Hemocytes are most likely the primary source of immune-related transcripts in our study. However, a supportive immune function by the pericardial cells in addition to their filtering activity may not be discarded. The immune capabilities of pericardial cells have been demonstrated on insects, with enrichment of immune signaling pathways and release of humoral components into the hemolymph (Cardoso-Jaime, et al. 2023; Meyer, et al. 2024). Immune markers have been detected in the pericardial cells of *Anopheles* mosquitoes (Barillas-Mury, et al. 1999; Danielli, et al. 2000; Levashina, et al. 2001). Moreover, mosquitoes exposed to yeast or zymosan display increased lysosomal activity in these cells (Hernández-Martínez, et al. 2013). However, the supportive role of these cells in immunity seems to be restricted to humoral processes, since they have been reported not to participate in cellular immune responses, such as phagocytosis, a function performed solely by the hemocytes (King and Hillyer 2012; Meier, et al. 2025). The extent to which *Cladomorphus* pericardial cells contribute to immune responses remains unclear and requires further investigation.

Transcripts related to the Toll and Imd signaling pathways are notably represented in our analysis, including Toll-like receptors, MyD88, NFKB and akirins (Cheung, et al. 2022; Goto et al. 2008; Horng and Medzhitov 2001; Lima, et al. 2021). These pathways are highly conserved across insect evolution and participate in pathogen-associated molecular patterns (PAMP) responsive signaling cascades, typically initiated by extracellular recognition events that culminate in the activation of intracellular effectors and synthesis of antimicrobial peptides (Lima, et al. 2021; Myllymaki, et al. 2014). Several Toll and Imd-related transcripts were reported to be enriched in the pericardial cells of *Drosophila* (Meyer, et al. 2024), suggesting that these cells may also contribute to immune signaling.

Antimicrobial peptides were also represented in the transcriptome, including pattern-recognition molecules (Beta 1,3 glucan binding proteins), modulators involved in antiviral response (pellino proteins), and antibacterial peptides (defensins, holotricins and lysozymes) (Hoffmann and Hetru 1992; Jensen 2023; Lee, et al. 1994; Manabe and Yamaguchi 2021). Lysozyme-like transcripts stood out as the most abundant antimicrobial category. These enzymes are usually induced upon bacterial infection, reaching high counts in the hemolymph (Hultmark 1996).

Melanization is involved in different defense mechanisms on insects, such as encapsulation, nodulation and wound healing, thus playing an important role in invader elimination and responses to foreign stimuli on insects (González-Santoyo and Córdoba-Aguilar 2012; Nakhleh, et al. 2017; Satyavathi, et al. 2014; Tang, et al. 2006). Our investigation pointed out numerous pigmented bodies around the heart, commonly surrounded by hemocytes. Moreover, the transcriptomic analysis indicates the expression of key components of the melanization response, including phenoloxidase-1, acting on the conversion of phenolic substrates to quinones, later forming melanin through polymerization, as well as other proteins involved in the melanization cascade, including activation factors (González-Santoyo and Córdoba-Aguilar 2012; Tang, et al. 2006). While the hemocytes are possibly the main source of such transcripts, pericardial cells may also contribute, as phenoloxidase was reported to be enriched in these cells on *Drosophila* flies (Meyer, et al. 2024). Taken together, our observations suggest physiological processes related to melanization around the heart of *Cladomorphus*, possibly mediated by hemocytes. However, immune assays, such as a bacterial challenge in the hemolymph, are necessary to fully elucidate the immune role of the heart region in *Cladomorphus*.

## Conclusion

Our structural data indicate that pericardial cells play a central role in controlling hemolymph composition in *Cladomorphus* through molecular uptake and lysosomal degradation, likely contributing substantially to endosome- and lysosome-related transcriptomic signatures, although hemocytes also participate in endocytosis and intracellular digestion. The heart and its associated cells emerge as a key site of immune defense, supported by the abundance of hemocytes and immune-related transcripts, particularly those involved in melanization and antibacterial responses. Overall, this study expands current knowledge of heart morphophysiology in Phasmida and highlights the *Cladomorphus* heart as a multifunctional system integrating circulation, homeostasis, and immunity, while leaving open the potential contribution of pericardial cells to innate immune responses.

## Supplementary Information

Below is the link to the electronic supplementary material.

ESM 1 (DOCX 3.81 MB)

ESM 2 (ZIP 4.54 MB)

ESM 3 (XLSX 4.74 MB)

ESM 4 (XLSX 2.00 MB)

ESM 5 (XLSX 0.98 MB)

ESM 6 (ZIP 84.0 MB)

ESM 7 (PDF 449 KB)

## Data Availability

The generated quantitative data from this study are available in Supplementary information.
