# Correction to: Integrated morphological analyses of *Cladomorphus phyllinus* and transcriptomic analysis of *Cladomorphus trimariensis* provide insights into the cardiac morphophysiology of stick insects (Phasmida: Phasmatidae)

**DOI:** 10.1007/s00441-026-04088-z

**Published:** 2026-07-02

**Authors:** Vinícius Cordeiro Rocha, Henrique Barbosa da Silva, Renata Cristina Barbosa, Gustavo Ferreira Martins

**Affiliations:** Departamento de Biologia Geral, Universidade Federal de Viçosa, Viçosa, Minas Gerais 36570‑900 Brazil

**Correction to: Cell and Tissue Research**

10.1007/s00441-026-04084-3

The authors regret that the version of Figure 7 that appeared in the original published article is incorrect.

The incorrect Figure 7 appears below.
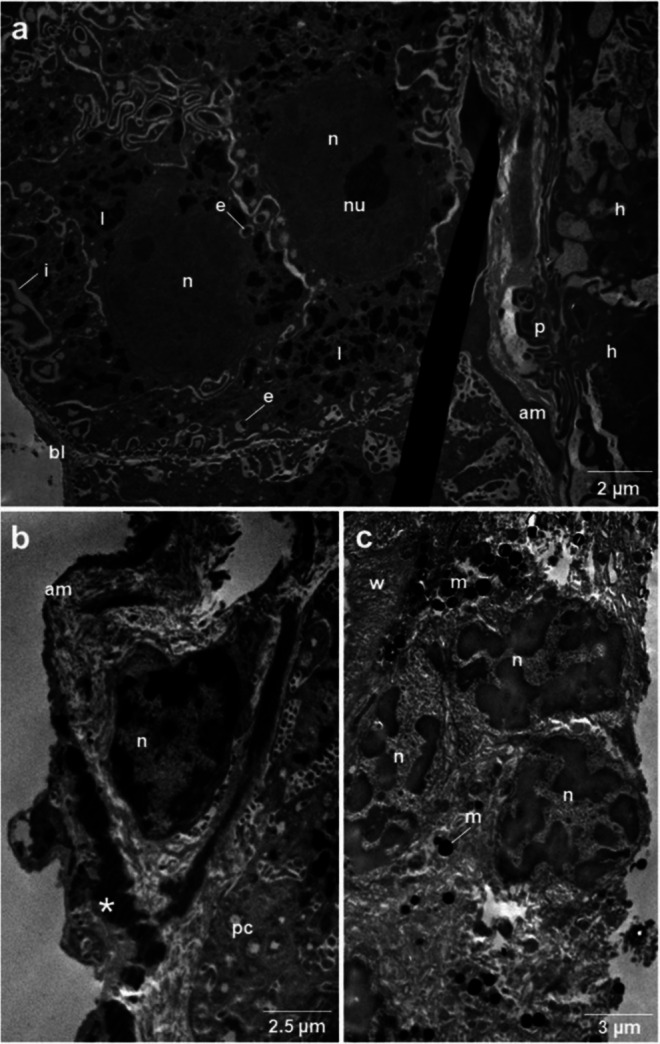


The correct Figure 7 appears below.
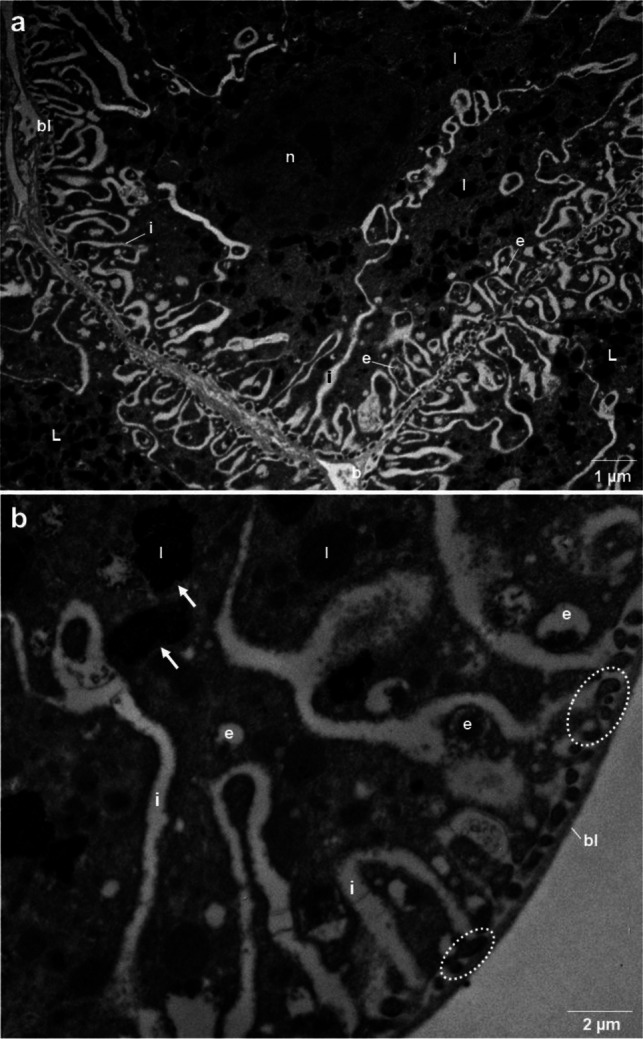


The original article has been corrected.

