# Supplementary material for: Integrated morphological analyses of Cladomorphus phyllinus and transcriptomic analysis of Cladomorphus trimariensis provide insights into the cardiac morphophysiology of stick insects (Phasmida: Phasmatidae)
Source: Cell Tissue Res. 2026 Jun 23;405(1):1. doi: 10.1007/s00441-026-04084-3 (PMC13287227; doi:10.1007/s00441-026-04084-3)
Supplement: Supplementary file 1 — (DOCX 3.81 MB) [file 441_2026_4084_MOESM1_ESM.docx]

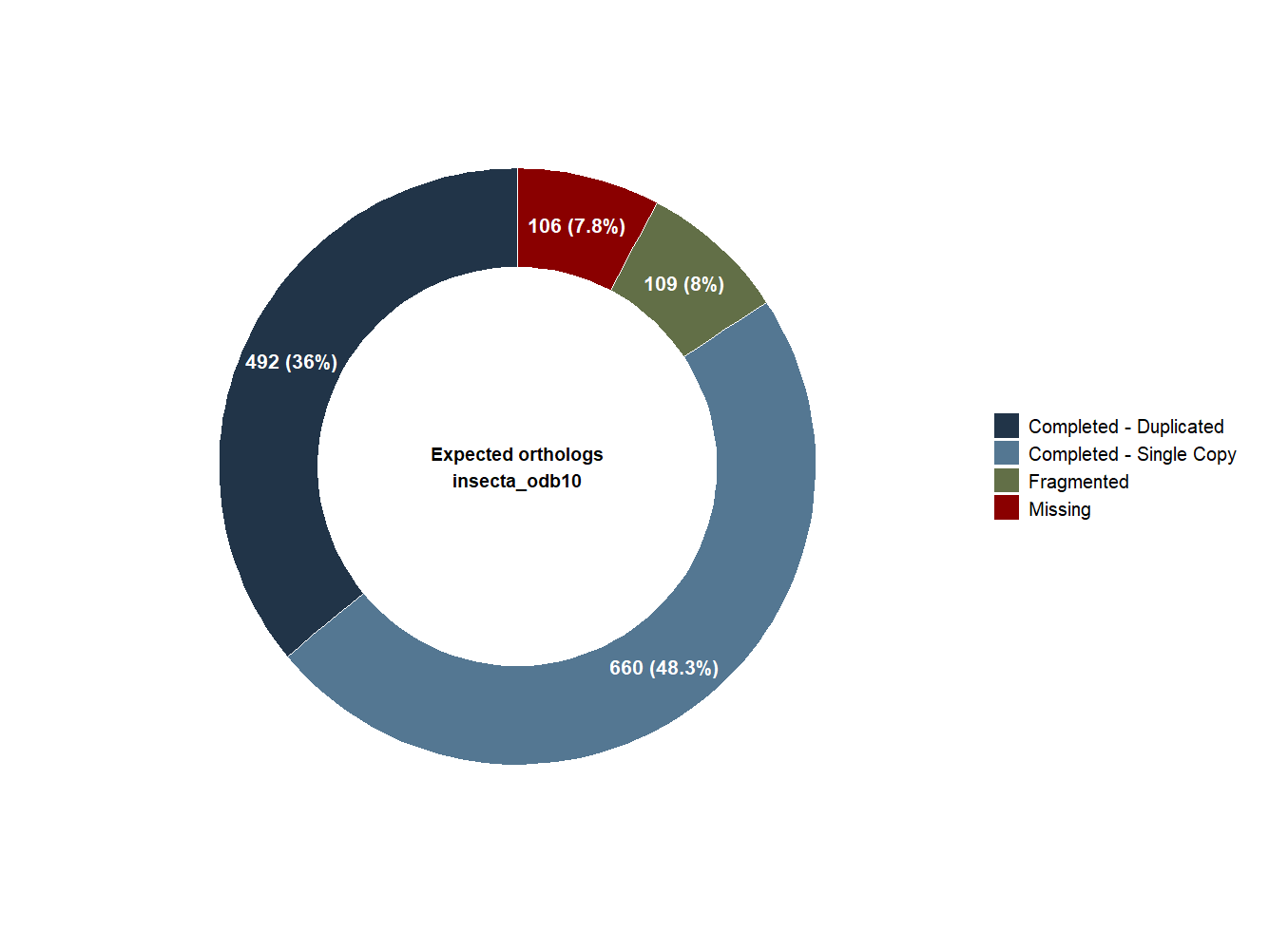


Analysis of transcriptome quality by Benchmarking Universal Single-Copy Orthologs (BUSCO) reporting 84.3% completeness, with completed genes being single-copy (48.3%) or duplicated (36%). A total of 8% of ortholog genes are fragmented and 7.8% are missing.
