# Supplementary material for: Integrated morphological analyses of Cladomorphus phyllinus and transcriptomic analysis of Cladomorphus trimariensis provide insights into the cardiac morphophysiology of stick insects (Phasmida: Phasmatidae)
Source: Cell Tissue Res. 2026 Jun 23;405(1):1. doi: 10.1007/s00441-026-04084-3 (PMC13287227; doi:10.1007/s00441-026-04084-3)
Supplement: Supplementary file 7 — (PDF 449 KB) [file 441_2026_4084_MOESM7_ESM.pdf]

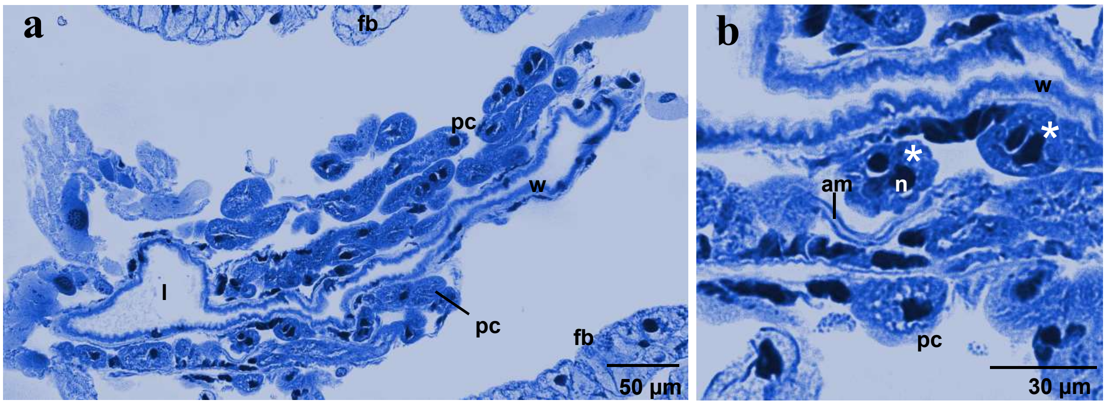

Fig. S1 Histological sections of the heart of *Cladomorphus trimariensis* third-instar nymphs stained with toluidine blue. **a** Portion of the nymphal heart showing pericardial cells (pc) adjacent to the muscular heart wall (w). fb: fat body. **b** Higher-magnification view of panel **a**, highlighting pericardial cells associated with branches of the alary muscles (am). *: binucleated pericardial cell; l: heart lumen; n: cell nucleus; w: heart wall.
